# Extracellular Monomeric and Aggregated Tau Efficiently Enter Human Neurons through Overlapping but Distinct Pathways

**DOI:** 10.1016/j.celrep.2018.03.021

**Published:** 2018-03-27

**Authors:** Lewis D. Evans, Thomas Wassmer, Graham Fraser, James Smith, Michael Perkinton, Andrew Billinton, Frederick J. Livesey

**Affiliations:** 1 Talisman Therapeutics, Babraham Research Campus, Cambridge CB22 3AT, UK; 2 Gurdon Institute and Department of Biochemistry, University of Cambridge, Cambridge CB2 1QN, UK; 3 AstraZeneca Neuroscience Innovative Medicines and Early Development, Granta Park, Cambridge CB21 6GH, UK

**Keywords:** Alzheimer’s disease, frontotemporal dementia, Tau, MAPT, iPSC, endocytosis, human neurons, intracellular transport

## Abstract

In Alzheimer’s disease, neurofibrillary tangle pathology appears to spread along neuronal connections, proposed to be mediated by the release and uptake of abnormal, disease-specific forms of microtubule-binding protein tau *MAPT*. It is currently unclear whether transfer of tau between neurons is a toxic gain-of-function process in dementia or reflects a constitutive biological process. We report two entry mechanisms for monomeric tau to human neurons: a rapid dynamin-dependent phase typical of endocytosis and a second, slower actin-dependent phase of macropinocytosis. Aggregated tau entry is independent of actin polymerization and largely dynamin dependent, consistent with endocytosis and distinct from macropinocytosis, the major route for aggregated tau entry reported for non-neuronal cells. Anti-tau antibodies abrogate monomeric tau entry into neurons, but less efficiently in the case of aggregated tau, where internalized tau carries antibody with it into neurons. These data suggest that tau entry to human neurons is a physiological process and not a disease-specific phenomenon.

## Highlights

• Extracellular tau protein enters human neurons by endocytosis and micropinocytosis
• Aggregated tau enters human neurons primarily by endocytosis
• Tau antibodies reduce uptake and are carried into neurons by tau
• Findings suggest that tau uptake is dependent on carrier proteins or receptors

## Graphical Abstract

**Figure:**
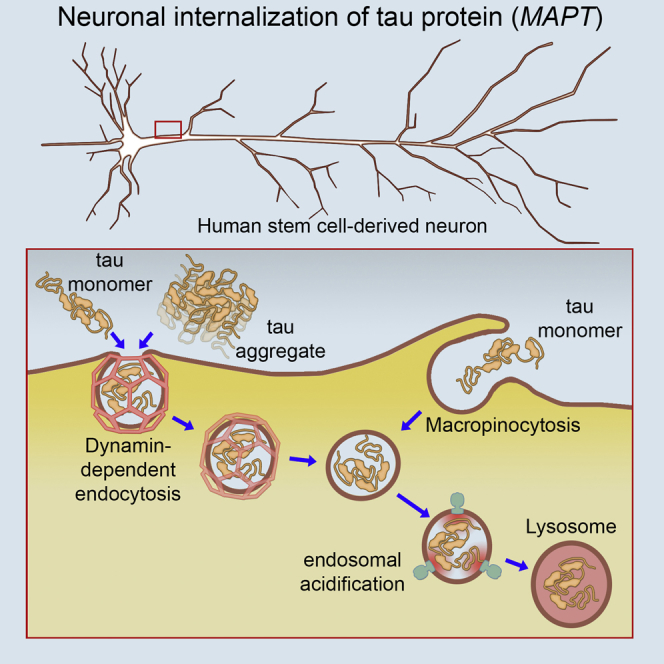

## Teaser

In contrast with predictions that transfer of the microtubule-associated protein tau between neurons is a toxic gain-of-function process in dementia, Evans et al. show that healthy human neurons efficiently take up both normal and aggregated tau, by distinct but overlapping uptake mechanisms.

## Introduction

The microtubule-associated protein tau (*MAPT*) is involved in the pathogenesis of several forms of dementia, including Alzheimer’s disease (AD) and frontotemporal dementia (FTD). Mutations in the *MAPT* gene are causal for some familial forms of FTD (Ghetti et al., 2015), and the formation of intracellular, hyperphoshorylated aggregates of tau (neurofibrillary tangles [NFTs]) is a common pathological feature in AD, FTD, and other dementias (Grundke-Iqbal et al., 1986, Kosik et al., 1986). Disease progression, and clinical severity of symptoms, is associated with a predictable spatial and temporal order of appearance of NFTs in different forebrain regions (Braak and Braak, 1991). This has led to the proposal that these diseases actively spread from diseased to healthy neurons in a spatial and temporal progression, mediated by extracellular, abnormal, disease-associated forms of tau (Iba et al., 2013, Liu et al., 2012).

It is not currently known if neuronal release and internalization of extracellular tau are disease-specific phenomena that spread disease through the CNS or are fundamental physiological processes. *In vivo*, tau protein is present in interstitial fluid of the CNS and passes into cerebrospinal fluid (CSF), where it is found in concentrations on the order of 15 pM (Blennow et al., 1995, Olsson et al., 2005). Mice transgenic for human tau have 10-fold higher concentrations of tau in brain interstitial fluid than in CSF, which would suggest that the extracellular concentrations of tau in the human brain are in the high picomolar-low nanomolar range (Yamada et al., 2011). There is strong evidence for the regulated release of non-pathogenic forms of tau from healthy neurons and of tau entry into neurons and non-neuronal cells (Bright et al., 2015, Chai et al., 2012, Kanmert et al., 2015, Wang et al., 2017). There have been conflicting reports about the ability of extracellular monomeric tau to enter cells, whereas aggregated or fibrillar tau has been clearly shown to efficiently enter neurons and other cell types (Frost et al., 2009, Kfoury et al., 2012, Wu et al., 2013).

We used human stem cell-derived neurons to address open questions about the efficiency with which tau enters human neurons, which forms (monomeric and aggregated) of tau enter neurons, and the routes by which they do so. We found that both forms of tau are efficiently taken up by human neurons, by overlapping but distinct mechanisms, consistent with regulated endocytosis. Monomeric tau enters neurons by two different routes and is actively trafficked within the neuron. Tau entry into neurons can be attenuated by antibody binding, however, extracellular tau-antibody complexes are internalized into intracellular compartments.

## Results

### Rapid and Efficient Entry of Extracellular Monomeric and Aggregated Tau into Human Neurons

We first analyzed the ability of monomeric and aggregated tau (P301S) proteins to enter human cortical neurons from the extracellular milieu. We used the tau P301S variant, an autosomal dominant mutation that causes early onset FTD with high penetrance (Bugiani et al., 1999, Guo et al., 2017). Focusing on P301S tau enabled us to study the transmission of a disease-relevant, aggregation-prone variant that differs from normal tau by a single amino acid, comparing monomeric and aggregated forms directly.

**Figure 1:**
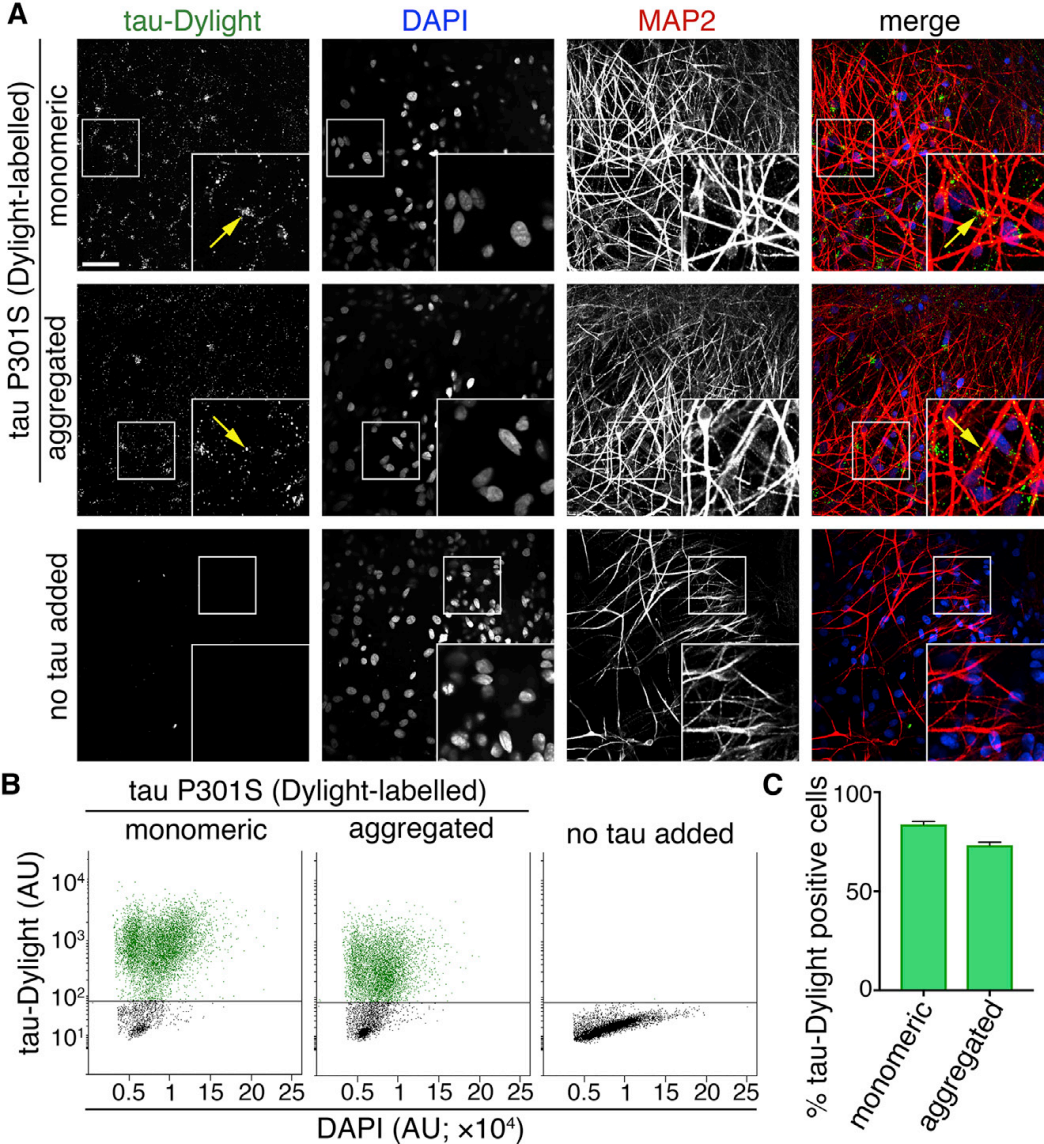
Human Neurons Efficiently Internalize Extracellular Monomeric and Aggregated Tau (A) Neuronal uptake of extracellular monomeric or aggregated forms of tau P301S protein conjugated to an amide-reactive fluorophore (green in merge; tau-Dylight) after 2 hr of incubation or without tau protein (no tau added). Fixed human iPSC-derived neurons (70 days after induction) were co-stained with DAPI (nuclear DNA; blue in merge) and MAP2 (neuronal dendrites and cell bodies; red in merge). Confocal microscopy representative images are shown. Scale bar, 50 μm. (B) Analysis of the proportion of tau-containing neurons following the addition of extracellular tau. Human iPSC-derived neurons (63 days after induction) were incubated with monomeric or aggregated tau-Dylight protein for 4 hr before dissociation into single cells and analysis by flow cytometry. Scatterplots of DAPI/tau-Dylight double-labeled neurons were gated by intensity of DAPI fluorescence and subsequently by tau-Dylight fluorescence. Neurons without tau incubation (no tau added) were used to establish the threshold level for detection of tau-Dylight fluorescence. (C) Percentage of DAPI/tau-Dylight double-positive iPSC-derived cells (total count of >7 × 10^3^ cells) following incubation with either monomeric or aggregated tau. Error bars indicate SEM (n = 6). See also Figures S1–S4.

To do so, purified recombinant monomeric (native) or heparin-aggregated tau proteins conjugated to an amide reactive fluorophore (tau-Dylight 488 NHS ester) were incubated with induced pluripotent stem cell (iPSC)-derived cerebral cortex excitatory neurons for 2 hr (Figure 1A; Figures S1 and S2). After extensive washing, monomeric and aggregated tau-Dylight were both detected within cells expressing the neuron-specific microtubule-associated protein MAP2, confirming that both forms of tau enter neurons. Tau-Dylight was found predominantly within the somatic compartment of neurons (Figure 1A). After a 4-hr incubation with extracellular tau, flow cytometry analysis (Figures 1B and 1C) revealed that 83% and 73% of dissociated cells contained monomeric or aggregated tau-Dylight, respectively, demonstrating that extracellular tau efficiently enters human neurons in culture. Internalization of monomeric tau (P301S) and wild-type tau was comparable and concentration dependent (Figure S3A), confirming that the P301S mutation does not confer the ability to efficiently enter neurons, nor is this form of tau likely to aggregate in extracellular media during the 3- to 4-hr incubation period. Tau derivatives and oligomeric states had no significant cytotoxic effect over the course of the assays (3–4 hr; Figures S3B and S3C).

### Concentration-Dependent Entry of Tau into Neurons via Low-pH Transport Vesicles

Live imaging of human cortical neurons was used to investigate the underlying mechanisms of neuronal entry of monomeric and aggregated tau. To do so, we prepared purified recombinant tau P301S protein conjugated to a maleimide-reactive pH-sensitive dye (tau-pHrodo Red), a reporter detectable in low-pH environments, including late endosomes and lysosomes. Incubation of tau-pHrodo with human neurons at a range of concentrations from 2.5 to 25 nM (0.12–1.2 μg.mL^−1^, diluted in culture medium) showed that tau entry to neurons is rapid, as visualized by live imaging. As we found with the amine-labeled tau-Dylight that conjugates the dye to lysine residues, maleimide-labeled tau-pHrodo (which conjugates to cysteine residues) showed concentration-dependent internalization, indicating that the position and chemical nature of each dye does not impact uptake.

To confirm that the dyes do not enable tau to enter neurons, we added FLAG-tagged tau with no fluorescent labels to human neurons, and we purified FLAG-tagged, intracellular tau from neurons at 24-hr intervals over a 4-day period using anti-FLAG immunoprecipitation. We found that FLAG-tagged tau enters neurons efficiently and that internalized tau persists at detectable levels within neurons for at least 4 days (Figure S4A). 3R and 4R splice isoforms of tau have one and two cysteines, respectively, enabling the production of forms of tau containing one or two pHrodo labels per tau molecule. We found that these two forms of pHrodo-tau differed approximately 2-fold in their fluorescent signal when added at the same concentration to human neurons, demonstrating the overall efficiency of protein labeling and the scaling of the fluorescent signal to intracellular tau entry (Figure S4B).

**Figure 2:**
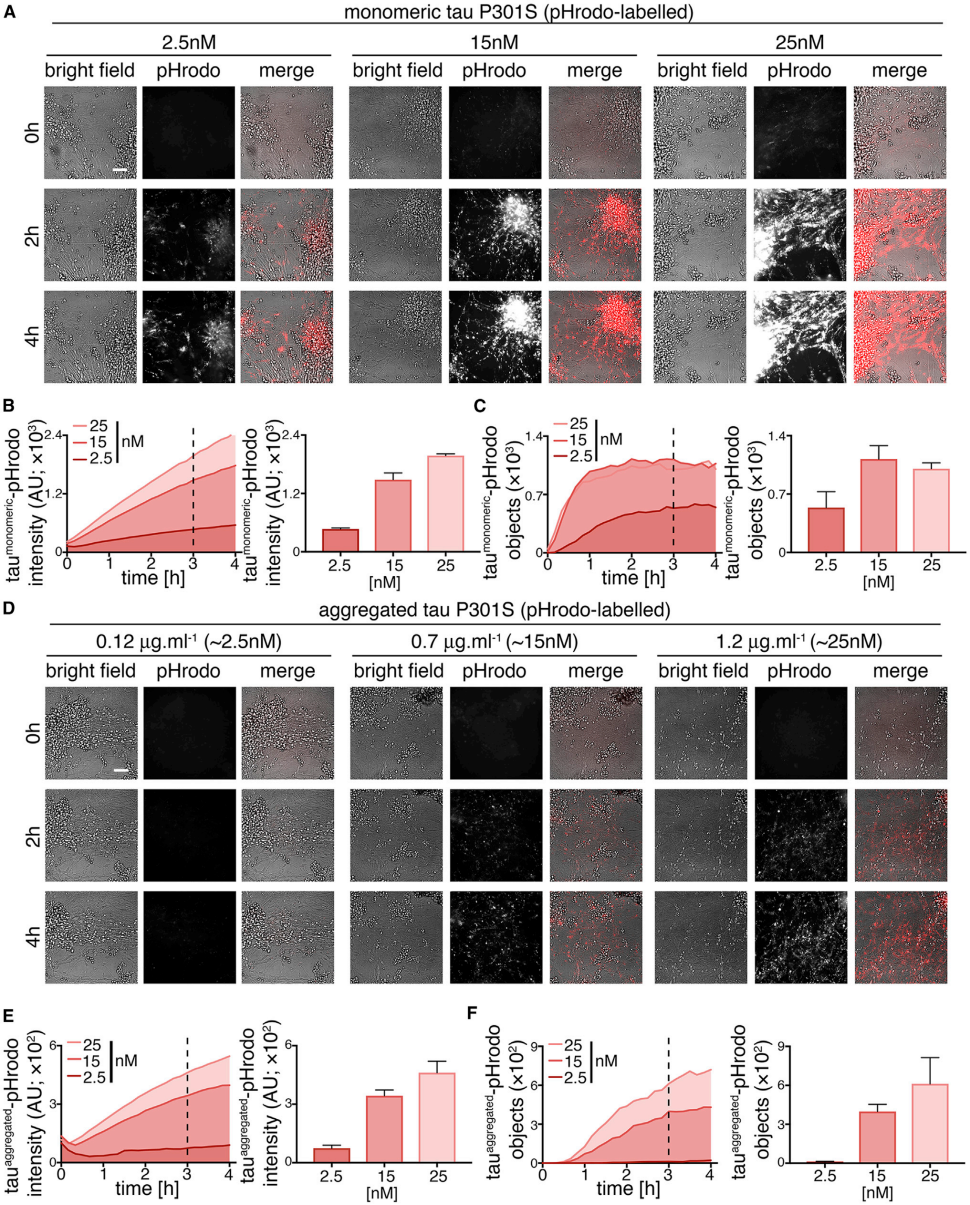
Human Neurons Internalize Extracellular Tau via Low-pH Intracellular Vesicles in a Concentration-Dependent Manner (A) Time-lapse (0- to 4-hr) images showing concentration-dependent uptake of extracellular monomeric tau P301S conjugated to a pH-sensitive dye (tau-pHrodo [2.5, 15, or 25 nM]; inverse relationship between fluorescence and pH) into iPSC-derived human neurons (75 days after induction). Bright-field (gray scale in merge) and pH-sensitive fluorescent signal (pHrodo; red in merge) were captured using automated imaging on the Opera Phenix platform (PerkinElmer). Nine independent measurements were taken from three technical replicates at 10-min intervals. Scale bar, 100 μm. (B and C) Following the extracellular addition of 2.5, 15, or 25 nM tau-pHrodo, the sum intensity of the pHrodo-positive objects (B) and the number of pHrodo-positive objects (C) were measured over 4 hr. Intensity (a.u.) and object measurements are displayed over time and at a 3-hr time point (dashed line); error bars indicate SD. (D–F) Concentration-dependent uptake of 0.12, 0.7, or 1.2 μg.mL^−1^ extracellular aggregated tau-pHrodo (equivalent to 2.5, 15, or 25 nM monomeric tau, respectively) into human neurons using the same experimental conditions and parameters as in (A). Sum intensity of the pHrodo positive objects (E) and the number of pHrodo positive objects (F) were measured as in (B and C). See also Figure S5.

Intracellular fluorescent punctae were observed within the first 10 min of exposure of neurons to monomeric tau-pHrodo (Figure 2A; Video S1). Tau-pHrodo-positive structures increased in size and intensity over the 4-hr course of the assay. These structures were present within neurites and accumulated in the cell bodies of neurons. In the presence of 15 and 25 nM monomeric tau-pHrodo, the number of tau-pHrodo-positive objects approached a plateau (>90% of final measurement) after approximately 1 hr (Figure 2C).

Internalization of aggregated tau-pHrodo (Figure 2D) was also found to be concentration dependent (Figure 2E). However, unlike monomeric tau-pHrodo, the number of detectable tau-pHrodo-positive objects (Figure 2F) did not increase immediately but displayed a lag for the first hour (Figure 2E). These kinetics of aggregated tau-pHrodo entry are similar to that of both lower concentrations of monomeric tau (2.5 nM) and of low-molecular weight (10-kDa) dextran-pHrodo (same molarity as monomeric tau samples; Figures S5A–S5C). Direct quantitative comparison of the amount of monomeric and aggregated tau-pHrodo entering at different concentrations is not possible under these experimental conditions, as the mixed nature of aggregated tau fibrils prevents us from establishing molarity accurately. We confirmed that heparin within the aggregated tau preparations did not contribute to tau entry into neurons, finding that uptake of both monomeric and aggregated tau was unaffected in the presence of heparin (Figures S4C and S4D).

### Monomeric Tau and Aggregated Tau Appear in Early Endosomes and in Lysosomes

**Figure 3:**
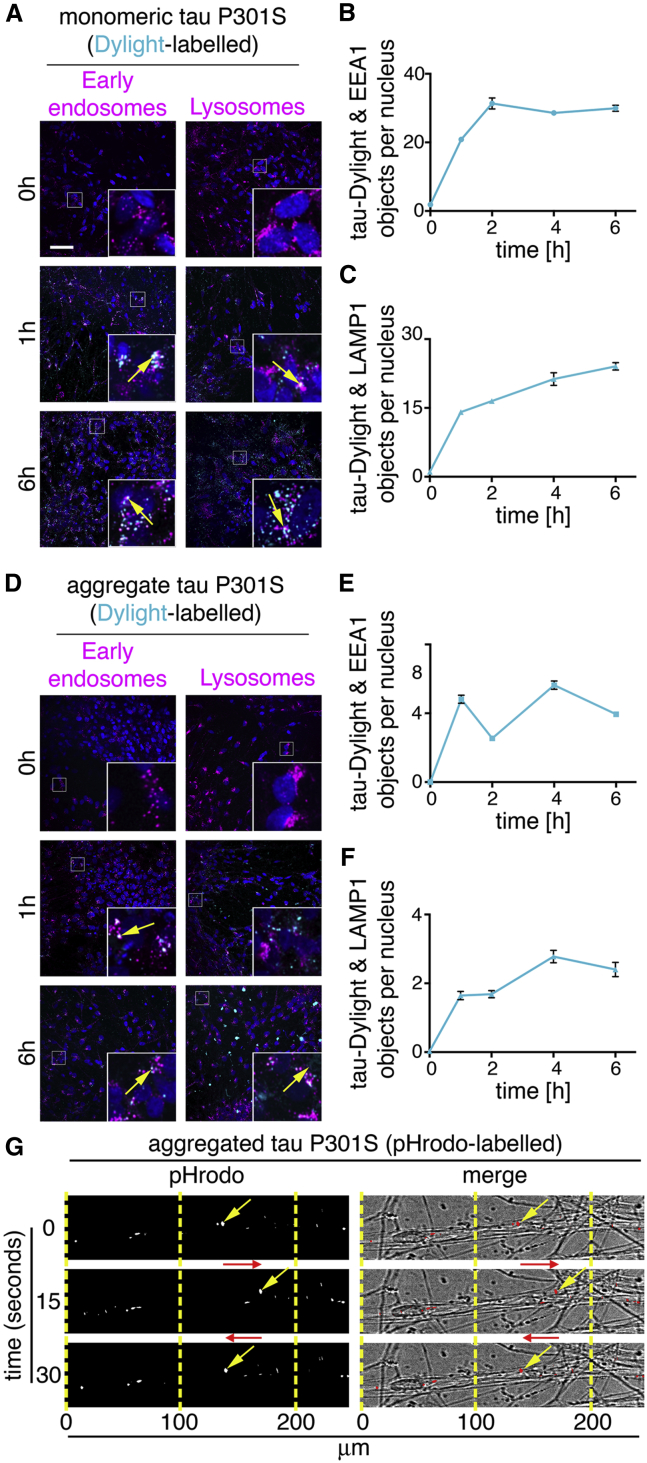
Internalized Monomeric and Aggregated Tau Are Trafficked in Early Endosomes and Lysosomes (A) Images of human iPSC-derived neurons (82 days after induction) following incubation with 100 nM monomeric tau P301S-Dylight (shown in cyan) for 0, 1, or 6 hr. Neurons washed with PBS were immunostained for EEA1 (early endosomes; left panel) or LAMP-1 (lysosomes; right panel) and nuclei were counterstained with DAPI (blue). Co-localization of tau-Dylight with endosomes and lysosomes appears white in images and is indicated by arrows; insets show magnifications of the area indicated in the main image. Scale bar, 50 μm. (B and C) Quantification of tau-Dylight and EEA1 (B) or LAMP1 (C) double-positive objects per nucleus over time. 25 independent z stacks were analyzed per condition and time point; error bars indicate SD. (D) Analysis of co-localization of internalized aggregated tau-Dylight (4.8 μg.mL^−1^, ∼100 nM equivalent of monomeric) with endosomes and lysosomes over 6 hr, using the same experimental conditions and parameters as (A). (E and F) Quantification of aggregated tau-Dylight and (E) EEA1- or (F) LAMP1- double-positive objects per nucleus over time. (G) Live tau-pHrodo imaging demonstrates the movement of low-pH vesicular structures in neurons. Representative images show neurons (80 days after induction) after 4-hr incubation with 1.2 μg.mL^−1^ aggregated tau-pHrodo imaged at 1-s intervals. Yellow arrows indicate intracellular mobile tau-pHrodo-positive objects and red arrows indicate the direction of movement. See also Videos S1 and S2.

To establish the route of vesicular internalization of monomeric and aggregated tau, we analyzed the appearance of tau-Dylight in endosomal or lysosomal compartments in iPSC-derived human neurons. Monomeric or aggregated tau-Dylight (total protein concentration 10 μg.mL^−1^, ∼250 nM) was incubated with neurons for up to 6 hr, then extensively washed to remove extracellular protein and fixed. Co-localization of tau with endosomes (early endosomal antigen-1 [EEA1] immunofluorescence; Figure 3A) or late endosomes and lysosomes (LAMP1 immunofluorescence; Figure 3B) was analyzed by confocal microscopy.

In agreement with the monomeric tau-pHrodo experiments, monomeric tau-Dylight was rapidly taken up into neurons (Figure 3C). In contrast, IgG-Dylight showed little detectable entry into neurons throughout the time course (data not shown). As early as 1 hr after the addition of extracellular tau, monomeric and aggregated tau-Dylight were co-localized in EEA1+ early endosomes. Monomeric and aggregated tau-Dylight were also detected in LAMP1+ late endosomes and lysosomes, consistent with endocytosed proteins first reaching early endosomes, before late endosomes and lysosomes.

Live imaging (Figure 3G; Video S2) of neurons incubated with aggregated tau-pHrodo (4 hr, 0.7 μg.mL^−1^) enabled the longer-term tracking of tau-pHrodo-containing vesicles within neurons. Tau-containing vesicles were rapidly (>10 μm/s) transported in both antero- and retrograde directions along neurites, and they could rapidly reverse direction of transport (Figure 3G). Thus, monomeric and aggregated tau both efficiently enter neurons via the endosome/lysosome system, and they are actively trafficked within vesicles over long distances within neurons over several hours.

### Differential Effects of Dynamin Inhibition on Endocytosis of Monomeric and Aggregated Tau

**Figure 4:**
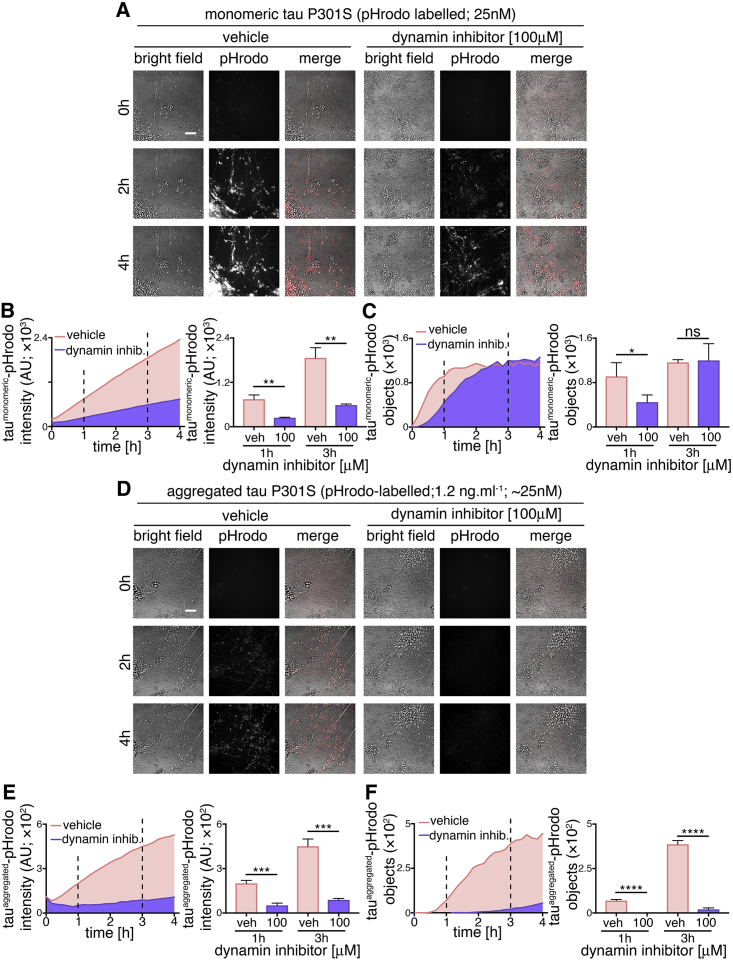
Monomeric and Aggregated Tau Enter Neurons via Different Mechanisms (A) Time-lapse (0- to 4-hr) imaging of human neuronal uptake of extracellular monomeric and aggregated tau P301S-pHrodo following dynamin inhibition. Neurons (75 days after induction) were pre-incubated with 100 μM Dynasore (dynamin inhibitor) or vehicle control (1% [v/v] DMSO) for 30 min prior to exposure to 1.2 μg.mL^−1^ extracellular tau-pHrodo and Dynasore or vehicle. Bright-field (gray scale in merge) and pH-sensitive fluorescent signal (pHrodo; red in merge) were captured using automated imaging on the Opera Phenix platform (PerkinElmer). Nine independent measurements were taken from three technical replicates at 10-min intervals. Scale bar, 100 μm. (B and C) Sum intensity of the pHrodo-positive objects (B) and number of pHrodo-positive objects (C) were measured over 4 hr. Intensity and object measurements are displayed over time and at 1- and 3-hr time points (dashed lines). Error bars indicate SD. Significance was determined using unpaired t tests (n = 3; ^∗^p < 0.05, ^∗∗^p < 0.005, ^∗∗∗^p < 0.0005, and ^∗∗∗∗^p < 0.0001). (D–F) Effect of dynamin inhibition on internalization and acidification of extracellular aggregated tau-pHrodo into intraneuronal compartments using the same experimental conditions and parameters as in (A). Sum intensity of the pHrodo positive objects (E) and the number of pHrodo positive objects (F) were measured as in (B and C). See also Figures S5 and S6.

As extracellular monomeric and aggregated tau utilize endosomal pathways to enter neurons but display different kinetics of uptake, we examined whether the underlying internalization mechanisms of tau protein species differ. First, we tested the sensitivity of monomeric and aggregated tau internalization to the small molecule inhibitor Dynasore (Figure 4), an inhibitor of the GTPase dynamin, which is required for numerous membrane fission events, including clathrin-mediated endocytosis (Preta et al., 2015). Following 30-min pre-incubation with 100 μM Dynasore or vehicle control, entry of extracellular monomeric (25 nM) or aggregated tau-pHrodo (1.2 μg.mL^−1^) was assessed by live imaging over 4 hr.

The amount of monomeric tau entering neurons, as measured by total fluorescent intensity of intracellular monomeric tau-pHrodo vesicles, was significantly reduced by dynamin inhibition, as shown at 1 and 3 hr after the addition of extracellular tau (Figure 4B). This was also reflected in the significant reduction in the number of tau-pHrodo-positive objects at 1 hr (compared with vehicle control; Figure 4C). The kinetics of appearance of intracellular monomeric tau-pHrodo objects changed in the presence of dynamin inhibitor, displaying an initial lag in entry. However, the number of tau-pHrodo objects then reached the same level as vehicle-treated controls by 3 hr, although not the same total intensity (Figure 4C), which may reflect a reduction in endosome/lysosome acidification in the presence of Dynasore. This suggests that there are two distinct mechanisms of entry of monomeric tau into human neurons, one of which is rapid and dynamin dependent and the other being slower and independent of dynamin.

The effect of dynamin inhibition on the entry of aggregated tau was more pronounced than on monomeric tau (Figures 4D–4F). The total fluorescent intensity of intracellular aggregated tau-pHrodo was consistently reduced by more than 70% at 1 and 3 hr after tau addition (Figure 4E), and the number of tau-pHrodo-positive objects was reduced by 95% (Figure 4F). These data indicate that, at the concentrations studied, aggregated tau enters neurons almost entirely via endocytosis.

### Perturbation of Actin Dynamics Confirms that Monomeric Tau and Aggregated Tau Have Overlapping but Different Routes to Enter Neurons

**Figure 5:**
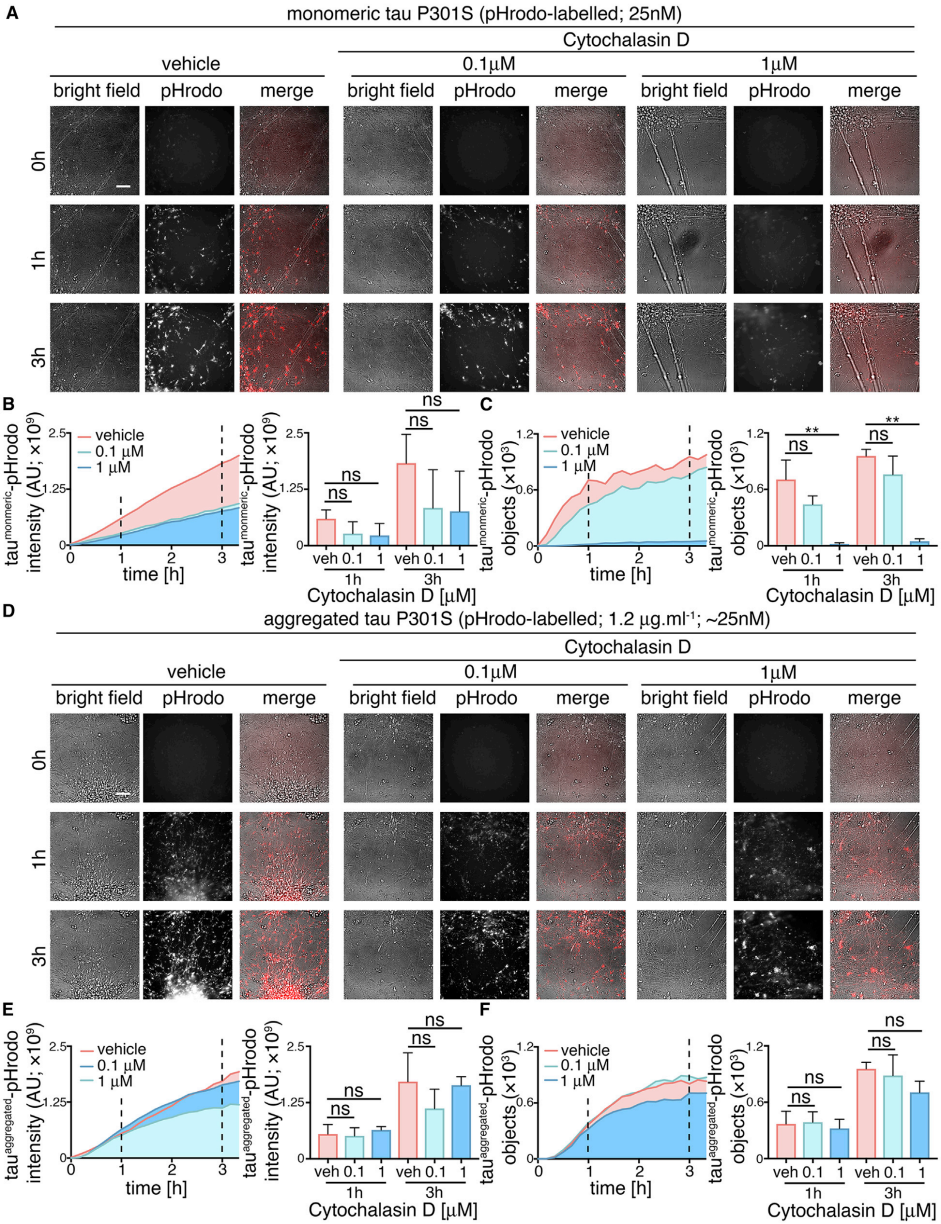
Perturbation of Actin Polymerization Confirms that Monomeric and Aggregated Tau Enter Neurons through Distinct Uptake Mechanisms (A) Time-lapse (0- to 3-hr) imaging of human neuronal uptake of extracellular monomeric and aggregated tau P301S-pHrodo following the inhibition of actin polymerization. Neurons (65 days after induction) were pre-incubated with 0.1 or 1 μM Cytochalasin D or vehicle control (1% [v/v] DMSO) for 30 min prior to exposure to 1.2 μg.mL^−1^ extracellular tau-pHrodo and Cytochalasin D or vehicle. Bright-field (gray scale in merge) and pH-sensitive fluorescent signal (pHrodo; red in merge) were captured using automated imaging on the Opera Phenix platform (PerkinElmer). Eight independent measurements were taken from three technical replicates at 10-min intervals. Scale bar, 100 μm. (B and C) Sum intensity of pHrodo-positive objects (B) and number of pHrodo-positive objects (C) were measured over 3.3 hr. Intensity and object number measurements are displayed over time and at 1- and 3-hr time points (dashed lines). Error bars indicate SD (n = 3; ^∗∗^p < 0.01, Dunnet’s multiple comparisons). (D–F) Effect of inhibition of actin polymerization on internalization of extracellular aggregated tau-pHrodo into low-pH compartments in human neurons using the same experimental conditions and parameters as in (A). Sum intensity of the pHrodo positive objects (E) and the number of pHrodo positive objects (F) were measured as in (B and C). See also Figures S5 and S6.

To explore further whether monomeric and aggregated tau enter neurons via different mechanisms, we studied the role of actin polymerization in this process (Figure 5). Inhibition of actin polymerization with Cytochalasin D disrupts several clathrin-independent endocytic processes, including bulk endocytosis/macropinocytosis (Mooren et al., 2012). Disruption of actin polymerization has previously been shown to inhibit entry of fibrils formed of the tau repeat domain (Holmes et al., 2013). Therefore, neuronal entry of tau-pHrodo was measured over 3 hr in the presence of extracellular monomeric (25 nM) or aggregated (1.2 μg.mL^−1^) tau-pHrodo, following a 30-min pre-incubation with 0.1 or 1 μM Cytochalasin D or vehicle control.

Entry of monomeric tau was markedly reduced in the presence of 1 μM Cytochalasin D, as reflected in the 95% reduction in the number of monomeric tau-pHrodo-positive objects after 3-hr incubation in the presence of 1 μM Cytochalasin D (Figure 5C). In contrast, disruption of actin polymerization with Cytochalasin D had little effect on the entry of aggregated tau (total fluorescent intensity and number of objects; Figures 5D–5F; Figure S5).

Live imaging of tau entry with pHrodo-tau may not detect intracellular pools of tau that enter via a different mechanism, avoiding low-pH environments. Additionally the pharmacological treatments may also affect the acidification of intracellular vesicles, which could affect the pHrodo signal. To control for this, we performed tau-Dylight internalization assays in the presence of 100 μM Dynasore or 1 μM Cytochalasin D (Figure S6), with 3 hr of incubation with 100 μM extracellular monomeric or aggregated tau. Compared with the live-imaging studies, relatively high concentrations of aggregated tau were used to investigate whether the dynamin-dependence and actin-independence of neuronal entry seen by live imaging were related to the lower molarity of aggregated versus monomeric tau used for those experiments. These independent assays confirmed the same differential effects of the two inhibitors observed by live imaging of pHrodo-tau: at the 3-hr assay point, dynamin inhibition had no effect on the number of monomeric tau-Dylight-positive punctae within neurons, whereas inhibition of actin polymerization reduced the amount of intracellular tau by over half (Figure S6). Conversely, dynamin inhibition significantly reduced the entry of aggregated tau, with no significant effects of Cytochalasin D (Figure S6) at this relatively high molar concentration of aggregated tau. The different pharmacological treatments and control recombinant proteins had no significant cytotoxic effect over the course of the assays (3–4 hr; Figure S5).

### Anti-tau Antibodies Slow Neuronal Internalization of Extracellular Tau

It is not currently known if tau entry into neurons requires specific domains of tau, specific cell surface-binding proteins, or receptors. Antibodies that recognize extracellular tau are a potential therapeutic strategy for slowing disease progression through the CNS by inhibiting tau entry into neurons (Congdon et al., 2016, Gu and Sigurdsson, 2011). Complex formation between specific antibodies and tau results in an increase in molecular size and may alter or mask uptake recognition sites on tau, thus preventing neuronal entry of tau.

**Figure 6:**
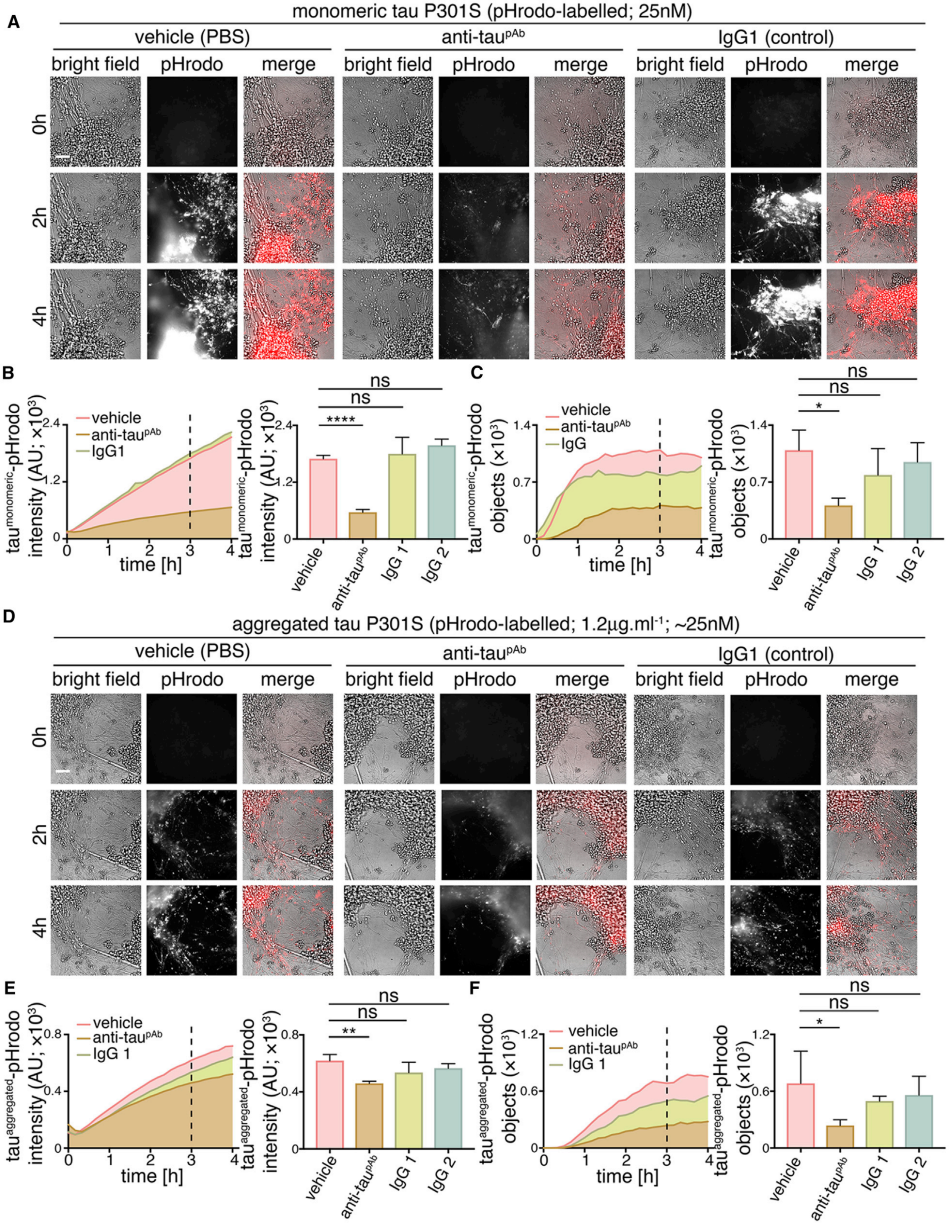
Anti-tau Antibody Attenuates Neuronal Uptake of Extracellular Tau (A–C) Time-lapse (0- to 4-hr) imaging of the effects of anti-tau polyclonal antibody on the internalization of extracellular monomeric tau-pHrodo by human neurons. Monomeric tau-pHrodo (25 nM) was pre-incubated with culture medium containing 250 nM polyclonal anti-tau (anti-tau^pAb^), IgG control (IgG1; IgG2) antibodies, or vehicle control (PBS) for 30 min. Tau-antibody incubations were added to iPSC-derived human neuronal cultures (75 days after induction). Bright-field (gray scale in merge) and pH-sensitive fluorescent signal (pHrodo; red in merge) were captured using automated imaging on the Opera Phenix platform (PerkinElmer). Eight independent measurements were taken from three technical replicates at 10-min intervals. Scale bar, 100 μm. (B and C) Intensity (B) and number (C) of pHrodo-positive objects measured over 4 hr after the addition of extracellular monomeric tau-pHrodo in the presence of vehicle, anti-tau polyclonal antibody, or two different control IgG antibodies. Intensity and object measurements are displayed over time and at a 3-hr time point (dashed line). Error bars indicate SD and significance was determined using one-way ANOVA (n = 3; ^∗^p < 0.05, ^∗∗^p < 0.005, and ^∗∗∗∗^p < 0.0001, Dunnet’s multiple comparisons). (D–F) Effect of tau antibody on the internalization of extracellular aggregated tau-pHrodo into human neurons using the same experimental conditions and analysis parameters as (A). Sum intensity of the pHrodo positive objects (E) and the number of pHrodo positive objects (D) were measured as in (B and C).

We analyzed the ability of a polyclonal antibody to the C-terminal half of tau to reduce tau protein entry into human cortical neurons. The anti-tau antibody or control IgGs (250 nM) were incubated with either monomeric (25 nM) or aggregated (1.2 μg.mL^−1^) tau-pHrodo at approximately 10-fold excess of antibody for 30 min. Tau-antibody mixtures were then added to iPSC-derived human neurons, and tau entry was assessed by live imaging of fluorescent intracellular tau-pHrodo (Figure 6).

Pre-incubation with the polyclonal (anti-tau^pAb^) tau antibody reduced the amount of monomeric tau that entered neurons, as assessed by the number of monomeric tau-pHrodo-containing vesicles (Figure 6B; Figure S7). The kinetics of monomeric tau entry were affected by the presence of tau-specific antibodies, such that an initial lag in the appearance of defined tau-pHrodo-positive objects was observed (Figure 6B).

Tau antibodies also reduced entry of aggregated tau uptake into human neurons (Figure 6C). In the presence of anti-tau^pAb^, the number of aggregated tau-pHrodo vesicles was reduced by more than half (Figure 6D; Figure S7). IgG antibodies that do not recognize tau appear to have some minor effects on monomeric and aggregated tau entry, which may be due to competition between tau and IgG for endocytosis.

The effect of tau antibodies on the entry of tau-Dylight into neurons was also tested (Figure S7), using the same concentrations and pre-incubation time as the tau-pHrodo assays. After 3 hr of incubation with antibodies and extracellular monomeric or aggregated tau, neurons were washed, fixed, and the number of intracellular tau-Dylight punctae was quantified. These independent assays confirmed that tau-specific antibodies significantly reduced the amount of tau-Dylight entering neurons (Figure S7).

### Entry of Aggregated Tau-Antibody Complexes into Neurons

**Figure 7:**
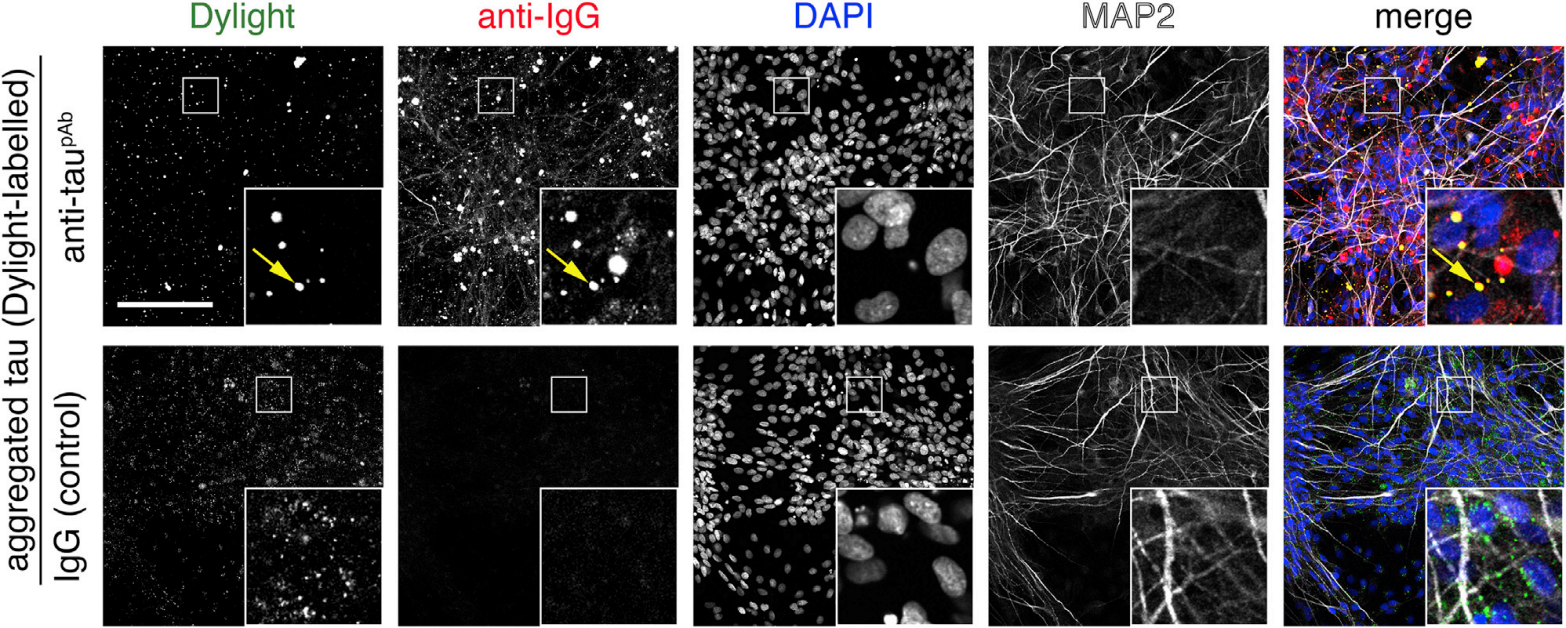
Aggregated Tau-Antibody Complexes Are Endocytosed by Human Neurons Confocal microscope images of neuronal uptake of extracellular aggregated tau P301S-Dylight-antibody complexes. Pre-formed (30-min incubation) aggregated tau-Dylight-antibody (anti-tau^pAb^ or an IgG control) complexes were added to iPSC-derived neurons (71 days after induction). After 3 hr of incubation, neurons were washed, fixed, and co-stained with antibody specific to the pre-incubation antibodies (donkey anti-mouse IgG Alexa 594 or donkey anti-rabbit IgG Alexa 594) and nuclear (DAPI; blue in merge) and dendritic markers (MAP2; white in merge). Scale bar, 100 μm. See also Figure S7.

Although tau was pre-incubated with a large molar excess of antibodies (10×) before addition to neurons, some monomeric and aggregated tau still entered neurons. This raised the question of whether the tau that entered neurons under these conditions did so in a complex with antibodies or if a fraction of antibody-free tau was available for neuronal entry. To distinguish between these possibilities, we performed additional assays using pre-formed aggregated tau-Dylight-antibody complexes. After 3 hr of incubation, antibodies were detected in the fixed neurons with a secondary antibody specific to the host species (anti-rabbit polyclonal; Figure 7) and also imaged for tau-Dylight. Intraneuronal punctae positive for both tau-Dylight and tau-specific antibodies were detected after binding of extracellular tau with anti-tau^pAb^, demonstrating that tau-antibody complexes do enter human neurons. No intraneuronal tau-antibody complexes were detected when aggregated tau was incubated with IgG control.

## Discussion

We find here that monomeric wild-type and FTD tau (P301S) and aggregated tau (P301S) all efficiently enter human neurons, arguing that tau entry to neurons is a constitutive biological process and not a disease-specific phenomenon. Both monomeric and aggregated species of tau enter neurons via a dynamin-dependent process, with monomeric tau also entering neurons through a second, slower pathway dependent on actin polymerization. Monomeric and aggregated tau access neurons via the lysosome/endosome system. Internalized tau persists within neurons for a minimum of 4 days, and a fraction of tau is dynamically transported long distances in neurons within low-pH vesicles for lengthy periods. The amount of monomeric and aggregated tau entering neurons is reduced by polyclonal anti-tau antibodies. However, a notable amount of tau enters neurons complexed with antibodies, even in the presence of a 10-fold molar excess of antibodies. While antibody binding may affect intracellular trafficking of tau and the final destination of tau within the neuron (McEwan et al., 2017), these data demonstrate that simple binding of tau by antibodies is not sufficient to prevent neuronal entry when tau is presented in solution to the neuron.

In contrast to earlier reports from HEK cells and primary rodent neurons (Frost et al., 2009, Holmes et al., 2013, Wu et al., 2013), full-length monomeric tau efficiently and rapidly enters healthy human neurons, and it does so as efficiently as aggregated tau. We find that there are two mechanisms of entry of monomeric tau into neurons: a rapid early phase that can be blocked by dynamin inhibition; and a second, slower phase that is actin dependent. These two mechanisms suggest that monomeric tau enters neurons via a rapid saturable dynamin-mediated endocytic mechanism and also by bulk endocytosis (Loebrich, 2014). In contrast to monomeric tau, aggregated tau entry is independent of actin polymerization and largely dynamin dependent, consistent with classic endocytosis. The lack of actin dependence of aggregated tau entry into neurons suggests that it does not enter human neurons via macropinocytosis, as has been reported in non-neuronal cells (Holmes et al., 2013).

Rapid endocytosis of both monomeric and aggregated tau would typically require one or more specific receptors or carrier molecules, the identities of which are currently unknown. Specific surface receptors have been identified for fibrils of alpha-synuclein (Mao et al., 2016), and that receptor shows specificity for aggregated synuclein over the monomeric form, which may suggest that, although both species of tau enter neurons via endocytosis, they may do so through distinct surface receptors. As a largely disordered protein, the most obvious candidate region for recognition for cellular entry of monomeric tau is the highly ordered microtubule-binding region of tau (Butner and Kirschner, 1991). Detailed understanding of the different paths of entry for monomeric and aggregated tau, and the regions of tau required for uptake, will be useful for investigating the potential for specifically preventing interneuronal transfer of aggregated tau without interfering with the transfer of non-pathogenic forms.

In addition to the importance of these questions for understanding mechanisms of dementia pathogenesis, they also have implications for immunotherapy strategies that target extracellular tau as disease-modifying treatments for dementia. Tau immunotherapy strategies have been shown to alter tau transmission in mouse models (Congdon et al., 2016, Gu and Sigurdsson, 2011). Proposed mechanisms for tau immunotherapies are epitope, affinity, conformation, and aggregate size dependent. Studies of specific tau immunotherapies have demonstrated that either extracellular (Yanamandra et al., 2013) or intracellular (Gu et al., 2013, Nicholls et al., 2017) tau can be targeted. Antibodies are thought to halt the progression of disease by binding to tau aggregates, triggering their uptake and clearance via either endosomal or proteasome pathways (Chai et al., 2011, Gu et al., 2013, Mallery et al., 2010). Fluid phase (Funk et al., 2015) and Fc receptor-mediated (Congdon et al., 2016) endocytosis have both been implicated in the uptake of antibody-tau complexes into neurons and microglia. The consequences for the neuron of uptake of antibody-antigen complexes are not clear, and they would depend in part on whether those complexes are degraded or accumulate in the endosome/lysosome or transit to the cytoplasm, where they could be detected by the cytosolic Fc receptor TRIM21 and targeted for degradation (McEwan et al., 2017).

The data presented here from *in vitro* experiments suggest that entry of tau into human neurons is an efficient physiological process rather than a disease-specific gain of function. Most current immunotherapy approaches targeting extracellular tau do not distinguish between pathogenic forms of tau that are thought to propagate disease and the forms of extracellular tau that are found in the healthy brain (Bright et al., 2015). Disruption of interneuronal transfer of non-pathogenic tau may have deleterious effects, if that transfer has a biological function, which is currently not known. Current clinical trials of anti-tau antibodies have not so far reported deleterious effects relating to the disruption of interneuronal transfer of non-pathogenic tau, and the field awaits the outcomes of longer-term trials. Furthermore, therapeutics that are not selective for extracellular pathogenic forms of tau may not achieve appropriate levels of target engagement in the presence of concentrations of non-pathogenic tau that are higher than those of pathogenic tau. Therefore, it will be important to ascertain the biological functions of neuronal release and internalization of tau. It will also be essential to identify which extracellular tau forms are truly pathogenic to facilitate the rational design of therapies that target interneuronal transfer of tau to prevent disease progression.

## Experimental Procedures

### Production and Characterization of Human iPSC-Derived Cerebral Cortex Neurons

Directed differentiation of human embryonic stem cells (hESCs) and iPSCs to cerebral cortex was carried out as described, with minor modifications (Shi et al., 2012a, Shi et al., 2012b). For drug treatment, all compounds were dissolved in DMSO at the concentrations noted, and DMSO was the vehicle control in all experiments. Compounds were added 30 min prior to incubation with recombinant tau protein (dynamin inhibitor Dynasore [Abcam] and actin polymerization inhibitor Cytochalasin D [Tocris Bioscience]). To establish identity and quality of neuronal induction, gene expression profiling was performed on a custom gene expression panel. RNA was isolated from iPSC cortical inductions, 35 days after induction, using an RNA extraction kit (QIAGEN), following the manufacturer’s instructions. Expression of genes in neurons (*MAP2*, *MAPT*, and *NGN2*), cerebral cortex progenitor cells (*EMX2*, *PAX6*, and *FOXG1*), ventral telencephalon (*NKX2-1* and *LHX8*), and mid-/hindbrain (*HOXA* and *HOXB*) was assessed in all neuronal inductions on the Nanostring nCounter platform. Cytotoxicity/cytolysis were assessed by analyzing the level of lactate dehydrogenase (LDH) activity (Roche) in conditioned media samples following treatments.

### Recombinant Tau Expression and Purification

Tau P301S_10×his-tag_avi-tag was overexpressed in BL21(DE3) bacteria. Cells were lysed using BugBuster (Millipore), and clarified lysate was applied to a 5-mL HisTrapHP column (GE Healthcare) in 2× PBS. Tau was eluted using a 0- to 500-mM imidazole gradient. Peak fractions were pooled and further purified in 2× PBS using a Superdex 200 16/60 gel filtration column (GE Healthcare). Pooled fractions were then concentrated to approximately 8 mg/mL using a spin concentrator (Millipore). Final protein concentration was determined by Nanodrop analysis.

### Preparation of Tau Oligomeric Species

1 mL tau P301S at 8 mg/mL was incubated with 4 mg.mL^−1^ heparin (Sigma) in PBS plus 30 mM 3-(*N*-morpholino)propanesulfonic acid (MOPS) (pH 7.2) at 37°C for 72 hr. Aggregated material was diluted in 9 mL PBS plus 1% (v/v) sarkosyl (Sigma) and left rocking for 1 hr at room temperature to completely solubilize any non-aggregated material. Insoluble tau was pelleted by ultracentrifugation for 1 hr at 4°C. The pellet was resuspended in 1 mL PBS by vigorous pipetting and sonicated at 100 W for 3 × 20 s (Hielscher UP200St ultrasonicator) to disperse clumps of protein and break large filaments into smaller species.

### Isolation of Internalized Recombinant Tau and Western Blot Analysis

Whole-cell protein extraction was performed by lysis of cell pellets in cell extraction buffer (Invitrogen) supplemented with PMSF (Sigma), protease inhibitors (Thermo Scientific), and phosphatase inhibitors (Thermo Scientific) before collection of the soluble fraction by centrifugation at 10,000 × *g* for 10 min. Soluble fractions were diluted in PBS and incubated with anti-FLAG M2 Magnetic Beads (Sigma) overnight; magnetic beads with bound proteins were washed following the manufacturer’s instructions and resuspended in lithium dodecyl sulfate (LDS) PAGE loading buffer. Western blot analysis was carried out using total tau antibody (Dako). Detection of immunoblot was carried out using LI-COR Odyssey CLx Infrared Imaging System and Image Studio Software.

### Labeling of Purified Recombinant Proteins

To label purified recombinant tau with Dylight 488 NHS ester (Thermo Fisher Scientific), monomeric or aggregated tau P301S (concentration of 8 and 2 mg.mL^−1^, respectively) was dialysed into PBS using Slide-A-Lyzer mini dialysis units (Thermo Fisher Scientific) overnight at 4°C. The dialysed tau preparation was then conjugated to 50 μg Dylight according to the manufacturer’s protocol. Non-incorporated dye was removed by overnight dialysis against 2× 1 L PBS at 4°C and the concentration determined using a bicinchoninic acid (BCA) protein quantification kit (Thermo Fisher Scientific).

To label purified recombinant tau with a pH-sensitive form of rhodamine (pHrodo, Thermo Fisher Scientific), 150 μM tau protein (or equivalent protein concentration for aggregate; ∼7 μg.mL^−1^) was incubated with 1.5 mM pHrodo Red Maleimide (dissolved in DMSO) and 1.5 mM tris(2-carboxyethyl)phosphine) (TCEP) (1:10:10 molar ratio, respectively) for 2 hr in the dark at room temperature (RT). After incubation, labeled protein samples were subjected to size exclusion chromatography at 4°C (Superdex 200 Increase 10/300 GL, GE Healthcare) in 50 mM phosphate (pH 7.4) and 150 mM NaCl to remove unreacted dye and assess perturbation of oligomeric state by labeling (no change was observed, data not shown). 10 mM 10-kDa dextran conjugated to pHrodo (Thermo Fisher Scientific) was dissolved in DMSO. 5 mg. mL^−1^ transferrin conjugated to pHrodo (Thermo Fisher Scientific) was dissolved in PBS.

### Analytical Size Exclusion Chromatography

Gel filtration chromatography used Superdex 200 increase 10/300 (GE Healthcare). Protein samples (0.1–5 mg.mL^−1^, 1% bed volume) were resolved at a flow rate of 0.5 mL.min^−1^; absorbance at 280 nm was monitored. Void volume and specific molecular weights of globular calibration proteins were determined using gel filtration low- and high-molecular weight calibration kits (GE Healthcare).

### Transmission Electron Microscopy

Formation of tau fibrils was confirmed by transmission electron microscopy, largely as described (Goedert et al., 1992). Briefly, samples were mounted on copper grids, stained with uranyl acetate, and negatively stained tau fibrils were imaged as described (Goedert et al., 1992).

### Labeled Tau Internalization Assays

Tau P301S labeled with either Dylight or pHrodo was diluted in neuronal maintenance tissue culture medium and added to neurons at the concentration and for the time indicated. Neurons were washed three times using 1× Hank’s balanced salt solution (HBSS) before fixation and processing for immunofluorescence and processing for flow cytometry or subjected to live imaging.

### Immunostaining, Confocal Microscopy, and Data Analysis

Immunohistochemistry was performed on neurons as previously described (Shi et al., 2012a) using the following primary antibodies: MAP2 from Abcam (ab5392), and EEA1 and LAMP1 from Cell Signaling Technology (C45B10 and D2D11). Secondary antibodies used were as follows: goat anti-mouse Alexa594 (A21125), goat anti-chicken Alexa 647 (A21449), goat anti-rabbit Alexa 546 (A11010), donkey anti-mouse Alexa 594 (A21203), and donkey anti-rabbit Alexa 594 (A21207, all from Thermo Fisher Scientific). Images were acquired using an Olympus FV1000 or Opera Phenix imaging systems. Typically conditions were repeated in triplicate and >12 images were collected per well. Quantification of fixed-cell imaging was performed using CellProfiler, using an automated pipeline. The pipeline first detected nuclei using the DAPI channel, their number, and the area was quantified. Next, the pipeline applied a fixed-intensity threshold that was applied to every image in the 488/tau channel to exclude low-level detector noise, followed by 488 positive tau object detection as primary objects and measuring of number and area. In co-labeling experiments, endosomes labeled with EEA1 or LAMP1 in the 546 channel were analyzed as described above for the tau channel. Next, a mask was created using the 546 channel. Application of this mask to the 488 channel allowed measuring of the number and area of tau objects covered by the mask, i.e., the number of tau-positive vesicles (objects) that overlap with endosomal markers EEA1 or LAMP1. To correct this measure for possible variations in cell density in the image, it was normalized to the number of nuclei.

### Flow Cytometry

After three 1× HBSS washes, neurons were dissociated using Accutase (Sigma, A6964) into single cells. Dissociated cells were collected by low-speed centrifugation (1,000 × *g* for 1 min), the supernatant was removed, and cells were fixed using 4% formaldehyde in PBS for 20 min at room temperature. Cells were washed three times with PBS, nuclei counterstained with DAPI, and analyzed using a BD LSRFortessa. Cells were gated using forward and side scatter channels to remove debris and gated using the side scatter channel to remove doublets. Fluorescence in the DAPI and 466 channels of this population was analyzed. Tau-negative neurons were defined using neurons that had not been treated with tau (indicated in black in Figure 1B) and tau-positive neurons (indicated in green). Flow cytometry data were analyzed using Flowing software.

### Live Imaging and Data Analysis

Neurons grown on μ-Plate 96 well (Ibidi) were imaged for fluorescent pHrodo-labeled protein (excitation at 561 nm and emission at 570–630 nm) in an acidic environment using a 40× confocal Opera Phenix high-content screening system (PerkinElmer). Bright-field and fluorescence emission images were collected at 10-min intervals. Parameters for fluorescent objects were set (fluorescent intensity and contrast) and quantified using the Harmony software (PerkinElmer). Data were analyzed using Prism Software (GraphPad). Typically, conditions were repeated at least in triplicate and <4 (typically 8) fields were recorded per well. To track individual objects, images were collected at 1-s intervals. Images were analyzed in Harmony 4.5 (PerkinElmer). Densely clumped areas of wells were excluded, and a spot-finding algorithm was used to detect foci of pHrodo-labeled protein. Within the spots, fluorescent intensity of the Alexa-568 channel was quantified representing the amount of labeled protein taken up into the endosomes. A high-intensity threshold was used with the spot-finding algorithm to detect only the very bright spots within the wells; discrete spots were counted to determine the number of pHrodo-positive vesicles within the cells.

### Statistical Analysis

The number of replica wells and experiments are indicated in the figure legends for each assay where appropriate. For live-cell imaging assays using pHrodo-labeled protein, intensity and number of objects were assessed as described in Live Imaging and Data Analysis. Typically, 8 fields were recorded per well and an average generated from the Harmony 4.5 software (PerkinElmer). Analyses were performed using the Prism version 7.0b software (GraphPad). One-way ANOVA with Dunnett’s multiple testing or Student’s t test was used where appropriate.

## Supplemental Information

Document S1. Figures S1–S7

Video S1. Human Neurons Take up Extracellular Monomeric Tau via Low-pH Intracellular Compartments in a Concentration-Dependent Manner, Related to Figure 2 Time-lapse (0–4 hr; × 2880 speed) video showing concentration-dependent internalization and acidification of extracellular monomeric tau^P301S^ conjugated to a pH-sensitive dye (tau-pHrodo [2.5, 15 or 25nM]) into iPSC-derived human neurons (75 days after induction). Bright field (gray scale) and pH-sensitive fluorescent signal (pHrodo; red) were captured using automated imaging on the Opera Phenix platform (Perkin Elmer).

Video S2. Extracellular Aggregated Tau Is Taken up and Trafficked Long Distances within Human Neurons in Low-pH Vesicles, Related to Figure 3 Time-lapse video (imaged at 1 frame/sec; × 2.5 speed) showing the movement of acidic vesicular structures containing tau in live human neurons (80 days after induction). Imaging performed after 4 hr incubation with 1.2μg.ml^-1^ of extracellular aggregated tau-pHrodo.

Document S2. Article plus Supplemental Information
