# Supplementary material for: Extracellular Monomeric and Aggregated Tau Efficiently Enter Human Neurons through Overlapping but Distinct Pathways
Source: Cell Rep. 2018 Mar 27;22(13):3612–24. doi: 10.1016/j.celrep.2018.03.021 (PMC5896171; doi:10.1016/j.celrep.2018.03.021)
Supplement: Document S1. Figures S1–S7 [file mmc1.pdf]

Cell Reports, Volume *22*

Supplemental Information

Extracellular Monomeric and Aggregated Tau Efficiently Enter Human Neurons through Overlapping but Distinct Pathways

Lewis D. Evans, Thomas Wassmer, Graham Fraser, James Smith, Michael Perkinton, Andrew Billinton, and Frederick J. Livesey

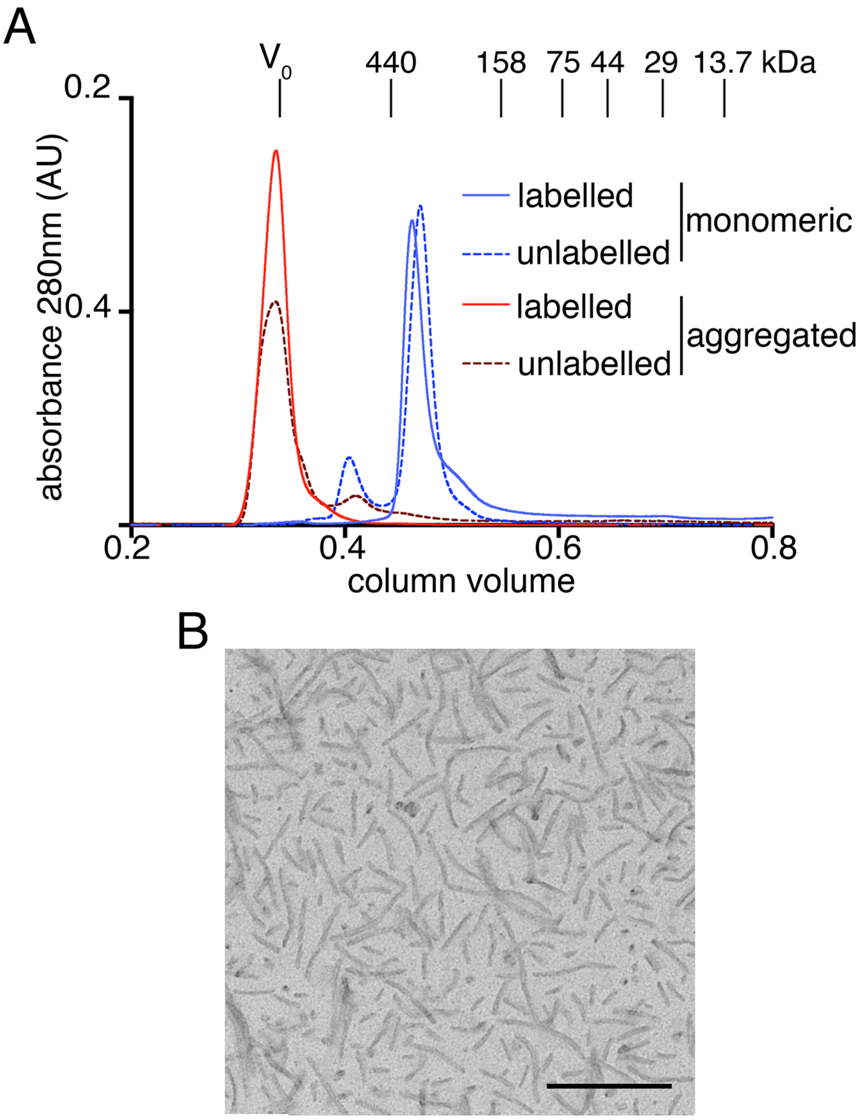

Supplemental Figure 1. Biophysical analysis of monomeric and aggregated forms of tau; relates to Figures 1 to 7

(A) Size exclusion chromatography (SEC) of purified labelled (solid lines) and unlabelled (dashed lines) for monomeric (blue) and aggregated (red) tau P301S. The void volume (V_0) and molecular weights (kDa) of globular calibration proteins are indicated at the top of the chromatography trace. (B) Transmission electron micrograph (TEM) of aggregated tau fibrils. Scale bar 500nm. Related to Figures 1 to 7 and Experimental Procedures describing analytical SEC and TEM.

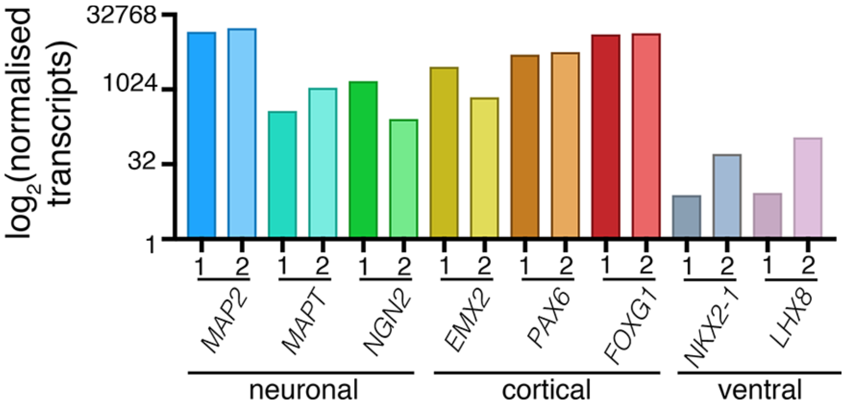

Supplemental Figure 2. Gene expression profile of human iPSC-derived neuronal inductions; relates to Figure 1

Number of RNA transcripts displayed on a log (base 2) scale from cortical neuronal progenitors and early stage neurons, 35 days after induction, normalised to housekeeping genes. Neuronal (*MAP2, MAPT* and *NGN2*), cortical (*EMX2, PAX6, FOXG1*) and ventral telencephalic (ventral); *NKX2-1, LHX8*) genes from two independent neuronal inductions (1,2). RNA transcripts for mid/hind-brain genes (*HOXA, HOXB*) were not detected (data not shown). Related to Experimental Procedures describing production and characterisation of human iPSC-derived cerebral cortex neurons.

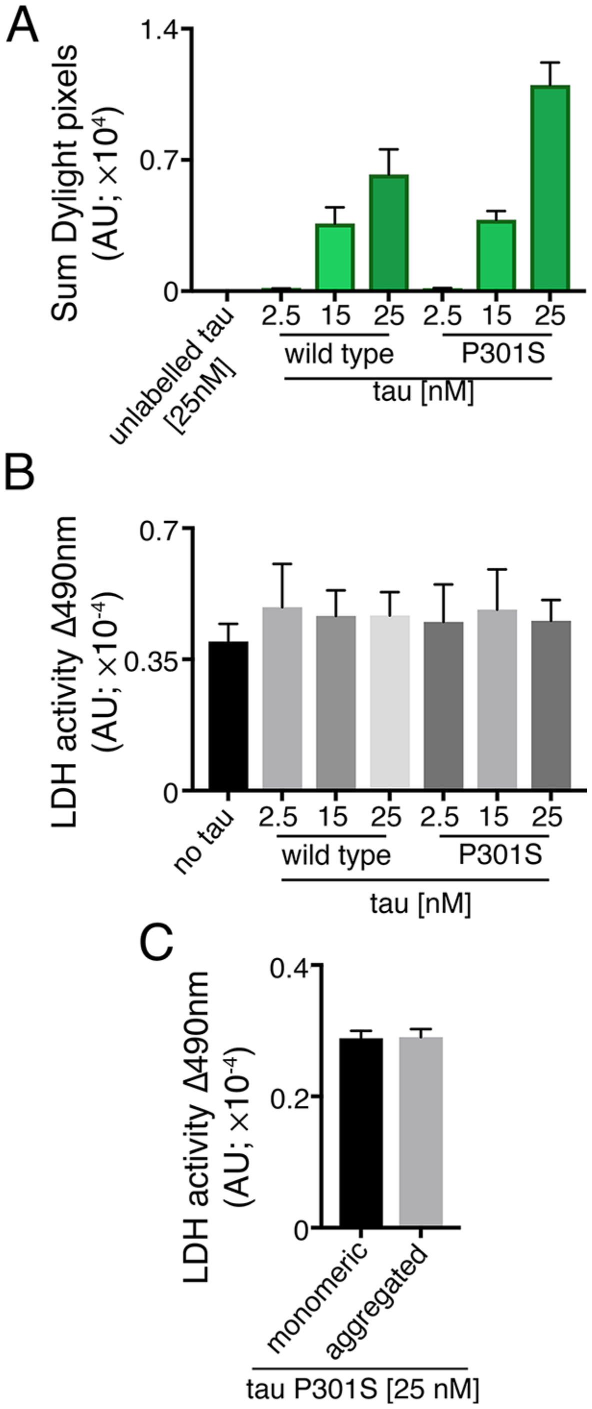

Supplemental Figure 3. Human neurons take up extracellular wild type and P301S tau in a concentration-dependent manner; relates to Figure 1

(A) Sum of Dylight-positive (488nm) pixels measured from confocal images of iPSC derived neurons (71-5 days after induction) incubated with 2.5, 15 or 25nM of either wild type or P301S tau protein with and without (unlabelled) conjugation to Dylight amine fluorophore (10 z-stacks; 9 fields; 3 replicate wells; 2 replica experiments). After 3 hours of incubation stem cell derived neurons were washed, fixed and co-stained with neuronal and nuclear markers (data not shown). The level of LDH activity in conditioned media, as measured by change in 490nm, was used to quantify neuronal cytotoxicity/cytolysis. (B) Extracellular LDH (measured by enzymatic assay) after extracellular addition of 2.5, 15 or 25nM of either wild type or P301S tau-Dylight protein or in the absence of recombinant tau (no tau; 71-5 days after induction, 3 replicate wells; 2 experiments). (C) Extracellular LDH after extracellular addition of 25nM monomeric and aggregated tau P301S (67-8 days after induction, 3 replicate wells; 2 experiments).

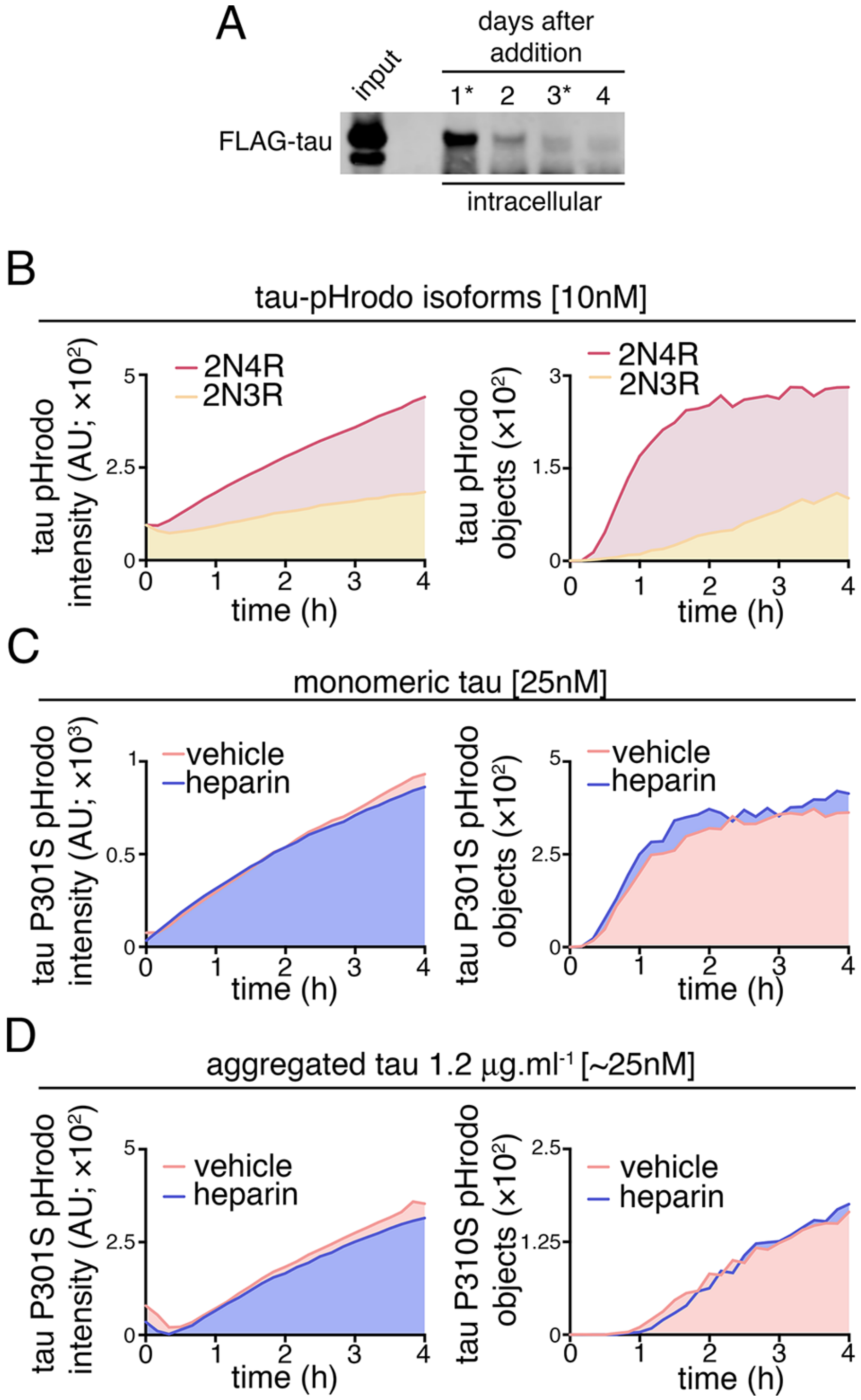

Supplemental Figure 4. Human neuronal uptake of tau is not dependent on dye labels or affected by free heparin; relates to Figure 2

(A) Time course (1-4 days) of purification and detection of FLAG-tagged isolated tau from soluble cell lysates (intracellular) following acute (24 hour) incubation with 50nM purified N-terminally FLAG×3 tagged tau (input). Cell culture media changes are indicated by '*'. (B) Time-lapse (0-4 hours) images assessing human neuronal internalization of malemide (cysteine reactive) labelled isoforms of tau protein. Neurons (90 days after induction) were incubated with culture medium containing 10nM of either 2N3R or 2N4R tau-pHrodo (containing one and two cysteine residues, respectively). Time-lapse (0-4 hours) images assessing human neuronal internalization of (C) monomeric and (D) aggregated tau-pHrodo in the presence of 1µM heparin. (B-D) Images were captured using automated imaging on the Opera Phenix platform (Perkin Elmer). Nine independent measurements were taken from three technical replicates at 10 minutes intervals. (Left panels) Sum intensity of the pHrodo positive objects and (right panels) number of pHrodo positive objects.

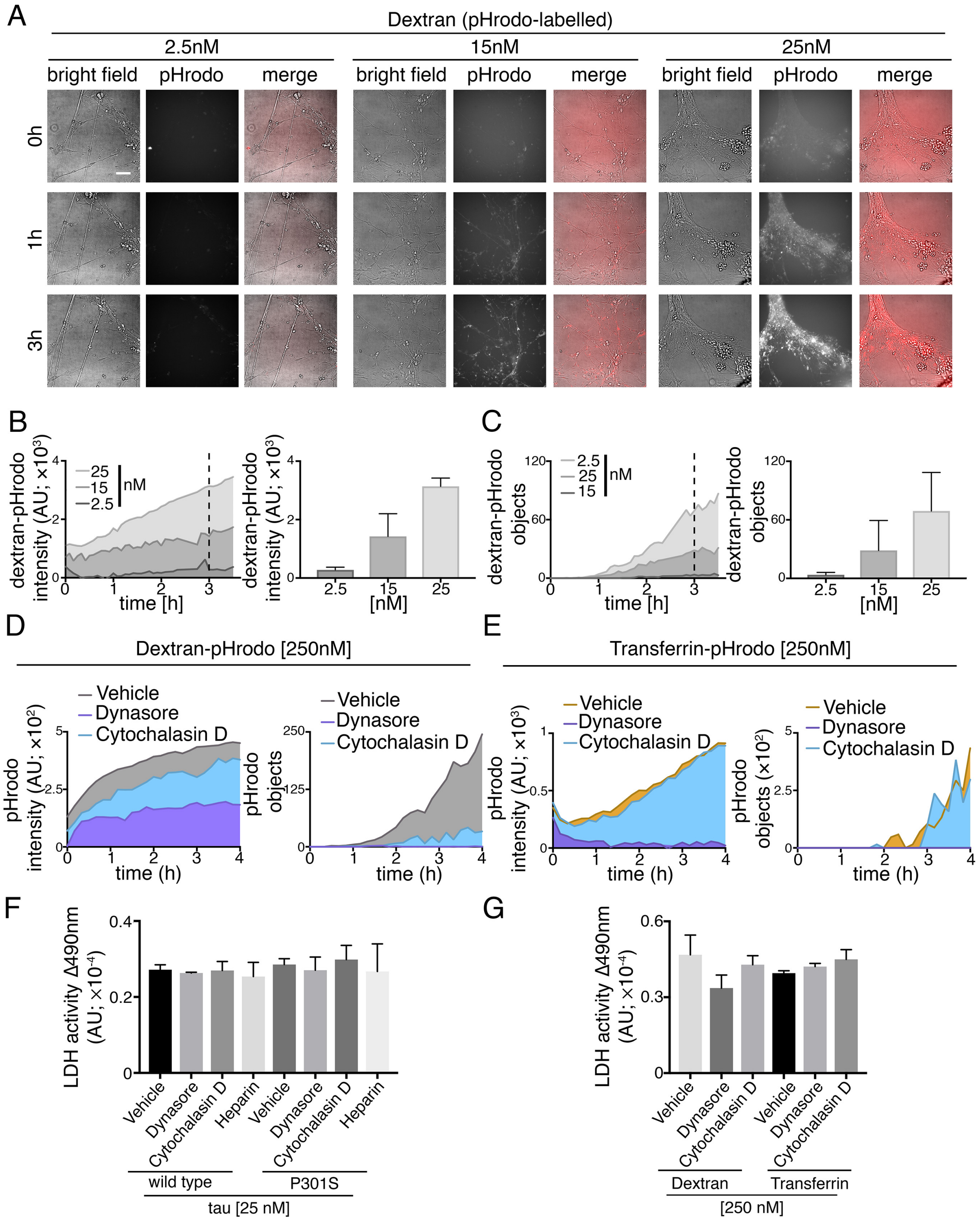

Supplemental Figure 5. Uptake of dextran or transferrin by human cortical neurons via low pH intracellular compartments in the presence of dynamin or actin polymerisation inhibitors; relates to Figures 2 and 4

(A) Time-lapse (0-4 hours) images showing concentration dependent uptake of extracellular 10kDa dextran conjugated to a pH-sensitive dye (dextran-pHrodo) by iPSC-derived human neurons. Bright field (grey scale in merge) and pH-sensitive fluorescent signal (pHrodo; red in merge). Four independent measurements were taken from three technical replicates at 5 minutes intervals. Scale bar 100μm. (B) Sum intensity of the pHrodo positive objects and (C) number of pHrodo positive objects. Intensity (AU) and object measurements are displayed over time and at a three-hour time point (dashed line). Validation of Dynasore and Cytochalasin D inhibitors acting on dynamin- and actin-dependent endocytic mechanisms, respectively. Internalization of 250nM extracellular (D) 10kDa dextran or (E) transferrin conjugated to a pH-sensitive dye (pHrodo) into human neurons (73 days after induction). Nine independent measurements were taken from three technical replicates at 10 minutes intervals (left and right panels are same metrics used in B and C, respectively). Extracellular LDH activity, in the presence of vehicle (0.5% DMSO), 100μM dynasore, 1μM cytochalasin D and 1μM heparin with (F) either 25nM wild type or P301S tau-pHrodo protein (3 replicate wells; 2 experiments) or (G) with 250nM transferrin or dextran (3 replicate wells). Error bars indicate standard deviation.

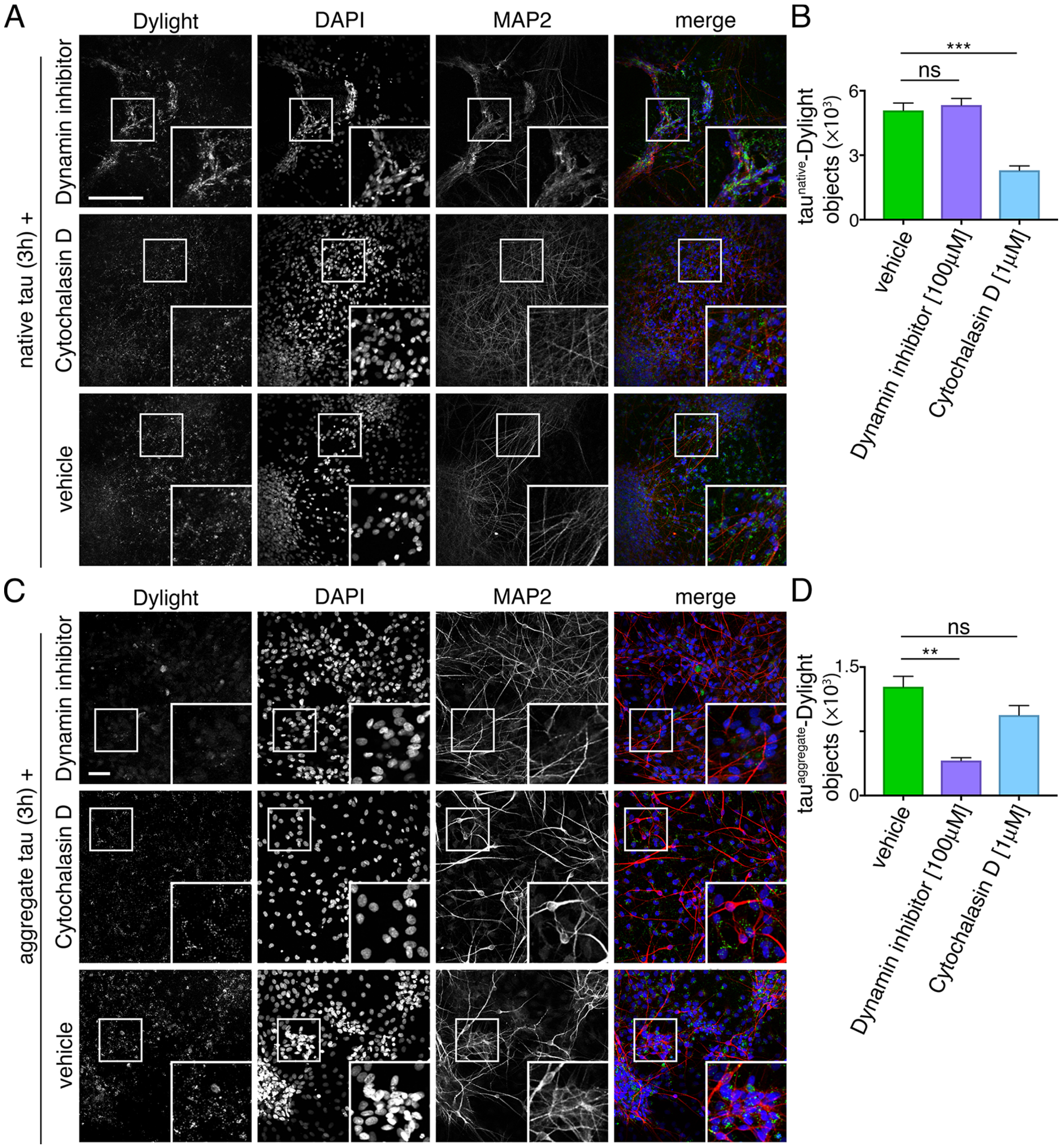

Supplemental Figure 6. Effect of Dynamin inhibitor or Cytochalasin D on tau-Dylight uptake by human cortical neurons; relates to Figures 4 and 5

(A) Confocal images of neuronal uptake of extracellular monomeric tau P301S protein conjugated to an amide reactive fluorophore (green in merge; tau-Dylight) after 2 hours of incubation with either dynamin inhibitor (Dynasore 100μM), Cytochalasin D (1μM) or with vehicle (DMSO). Fixed iPSC derived neurons (71 days after induction) were co-stained with DAPI (blue in merge) and MAP2 (dendrites and cell bodies; red in merge), respectively. 12 independent measurements were taken from three technical replicates. Scale bar 100μm. Fluorescent punctae of monomeric tau P301S (Dylight-labelled) were quantified (B). Error bars indicate s.e.m and significance was determined using one-way ANOVA (n = 3, ** = p<0.005, *** = p<0.0005 Dunnet's multiple comparisons). (C,D) Effect of Dynamin inhibitor or Cytochalasin D on extracellular aggregated tau (Dylight-labelled) for 3 hour internalisation. Scale bar 50μm.

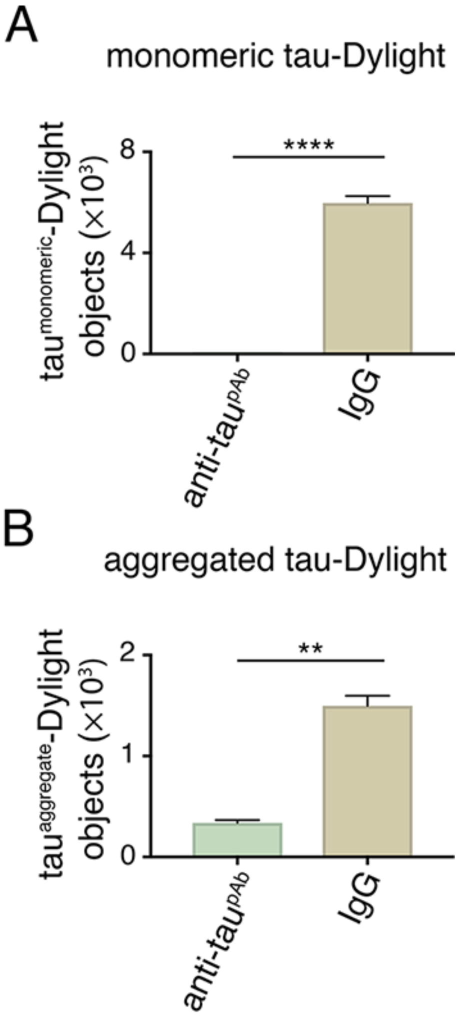

Supplemental Figure 7. Effect of pre-incubation of anti-tau antibodies with extracellular tau on uptake of Dylight-tau by human neurons; relates to Figure 7

Neuronal tau uptake experiments performed using 1.2 µg.ml-1 of (A) monomeric or (B) aggregated tau P301S (Dylight-labelled). Pre-incubated (30 minutes) monomeric or aggregated tau (Dylight-labelled) with either a tau specific antibody (anti-taupAb) or an IgG control were added to iPSC-derived neurons (71 days after induction). After three hours of incubation, neurons were washed and fixed. 12 independent measurements were taken from three technical replicates. Fluorescent tau P301S punctae (objects) were quantified, error bars indicate s.e.m. and significance determined using unpaired t test (n = 3, ** = p<0.005, **** = p<0.0001).
